# Exploring Cause of Death for Patients who Initially Present with Primary Progressive Apraxia of Speech

**DOI:** 10.1002/alz70858_100233

**Published:** 2025-12-25

**Authors:** Rene L Utianski, Gabriela Meade, Joseph R Duffy, Jennifer L. Whitwell, Hugo Botha, Keith A. Josephs

**Affiliations:** ^1^ Mayo Clinic, Rochester, MN, USA; ^2^ Department of Radiology, Mayo Clinic, Rochester, MN, USA; ^3^ Department of Neurology, Mayo Clinic, Rochester, MN, USA

## Abstract

**Background:**

Primary progressive apraxia of speech (PPAOS) is a frontotemporal lobar degeneration spectrum disorder characterized by progressive speech motor planning/programming decline. Patients often develop neurological symptoms, frequently evolving into progressive supranuclear palsy (PSP)/corticobasal syndrome (CBS) overlap syndromes. Requests for information about disease progression and causes of death are common amongst our clinical and research encounters; however, empiric evidence to guide these discussions is lacking. Understanding causes of death may enhance counseling and support targeted interventions to delay fatal outcomes. This study aimed to describe causes of death in PPAOS.

**Methods:**

Data from 29 deceased PPAOS patients (16 males) seen in research studies (July 2010–May 2023) were reviewed. Causes of death were established from medical charts and family reports. Fifteen had prosodic and 12 had phonetic‐predominant PPAOS; two had no clear predominance. Median age at onset was 67.08 years, disease duration 9.78 years, and age at death 76.25 years. 28 patients were white and one was Asian Indian.

**Results:**

Overall, fifteen (52%) patients died from overall deterioration (cachexia/dehydration), nine (31%) from aspiration pneumonia, and the remainder from falls (10%), asphyxiation (3%), or medical aid in dying (3%). There was no clear association with AOS subtype and cause of death.

**Conclusions:**

Most deaths in PPAOS resulted from systemic failure or dysphagia‐related complications. Half of the patients died without a clear precipitating event, described by family members as “shutting down,” similar to other prior studies that have shown central nervous system deterioration associated with dementia as cause of death. Aspiration pneumonia caused 34% of deaths, highlighting the need for early swallowing management, including assessments, dietary adjustments, and caregiver education. Falls underscore the importance of addressing balance and motor impairments through proactive prevention strategies. Other factors, such as access to healthcare and medical and psychiatric comorbidities were not analyzed but may influence outcomes. Future research should validate these findings in larger, diverse cohorts and compare outcomes to motor‐onset presentations of PSP and CBS. A comprehensive, multidisciplinary approach addressing motor speech impairments, dysphagia, and fall risks is essential to improve care and potentially extend survival for patients with PPAOS.

